# Comment on Dashtipour et al. Dysphagia and Muscle Weakness Secondary to Botulinum Toxin Type A Treatment of Cervical Dystonia: A Drug Class Analysis of Prescribing Information. *Toxins* 2024, *16*, 442

**DOI:** 10.3390/toxins17040190

**Published:** 2025-04-11

**Authors:** Richard Trosch, Daniel Parreirinha, Susanne Proeschel

**Affiliations:** 1 The Parkinson’s and Movement Disorders Center, Farmington Hills, MI 48334, USA; 2 Ipsen, Wrexham LL13 9UF, UK; daniel.parreirinha@ipsen.com; 3 Ipsen, 81677 Munich, Germany; susanne.proeschel@ipsen.com

We read with interest and some concern the article by Dashtipour and colleagues [1] and believe there is a need for a greater level of scientific scrutiny and context for the claims made in the paper.

Firstly, as your readers will no doubt be aware, comparisons between medications should only be performed within a single study and not across discordant studies utilizing different study methodologies. Direct comparisons across different studies are not scientifically valid. Secondly, the publication lacks a reference supporting the amount of nanograms of toxin present in daxibotulinumtoxinA (daxiBoNT-A). The comparison of toxin amounts in nanograms is invalid, as there is no single data set referenced that provides a side-by-side comparison using the same analytical assay and reference standards, as performed in Field and colleagues (2018), where onabotulinumtoxinA (onaBoNT-A), abobotulinumtoxinA (aboBoNT-A), and incobotulinumtoxinA (incoBoNT-A) were compared [2]. Accurate measurement by ELISA requires all determinations to be evaluated against the same reference standard preparation, within the same assay, and with powered testing. This approach minimizes assay-driven variability and allows for direct comparison. We also want to highlight that the publication compared a range of doses for incoBoNT-A and aboBoNT-A, while the authors compared the average dose for onaBoNT-A to daxiBoNT-A. Recent data presented at the Movement Disorders Congress suggest that a majority (65%) of patients treated with daxiBoNT-A in routine practice received doses above the 250 U maximum dose tested in the pivotal studies. More information is required at higher doses because adverse events are often dose-related.

The same logic applies to the clinical studies reviewed, which have used different study protocols, study populations, and injection techniques. While Dashtipour and colleagues noted that use of pivotal clinical trial data is a strength of their approach [1], it should be noted that the aboBoNT-A prescribing information includes safety data from the very earliest studies for aboBoNT-A (i.e., pre-phase 3) [3]. In the decades since the first clinical studies, our understanding of how to best inject for cervical dystonia has considerably advanced—and the investigators involved in the design and execution of the daxiBoNT-A trials have only benefited from this long experience. For example, unlike the recent daxiBoNT-A study [4], the dose-finding study reported by Poewe et al. (1998) did not exclude participants with pre-existing dysphagia and importantly found no difference in the rates of dysphagia with aboBoNT-A versus placebo [5]. Note that the publication did not consider placebo groups as part of the analysis [1]. For this early study, the 1000 U dose of aboBoNT-A (with 300 U into the sternocleidomastoid [SCM]) was chosen to test the safety and efficacy of the upper end of an empirically defined dosing range, as there were no good data previously available to base dosing on [5]. Later analysis of the aboBoNT-A database showed that unilateral injections of >150 U into the SCM are associated with a higher dysphagia risk [6], and the US prescribing information clearly notes that limiting the dose injected into the SCM may reduce the occurrence of dysphagia [3]. Indeed, as noted in the US prescribing information, the upper 75th percentile of SCM dosing in the phase 3 studies was 150 U [3]. Moreover, we have since learned that the use of injection guidance, particularly ultrasound, is important for the accurate injection of this long but thin muscle to reduce dysphagia [7]. We do not know how many participants were injected under injection guidance in the 2024 daxiBoNT-A study [4], but we do know that none were injected with guidance in the 1998 aboBoNT-A study [5].

While the pivotal studies may be old, there is a wealth of safety data available to inform injectors on best practice with the established BoNT-A products. For example, in a meta-analysis of three open-label studies including 1202 patients treated with aboBoNT-A in routine practice, the median dose was consistently 500 U (median dose of 130 U in the SCM; 38% under injection guidance) [8]. Safety data were available for two of those observational studies, and the rates of muscle weakness (1.2–3.7%) and dysphagia (1.7–6.8%) were often related to higher doses in the SCM [9,10] and were much lower than indicated in the Dashtipour analysis [1].

The beauty of clinical science is that it always moves forward and builds on prior knowledge gained by the pioneers of the field. We reiterate the scientific convention that direct comparisons should only be made within a study. To correctly assess the relative safety between different toxin products, a single, head-to-head study should be designed to compare the rate of adverse effects at doses that achieve similar clinical benefits.
